# Treatment outcome comparisons of first‐line targeted therapy in patients with *KRAS* wild‐type metastatic colorectal cancer: A nationwide database study

**DOI:** 10.1002/cam4.6196

**Published:** 2023-06-16

**Authors:** Yi‐Hsin Liang, Kuo‐Hsing Chen, Yu‐Yun Shao

**Affiliations:** ^1^ Graduate Institutes of Oncology National Taiwan University College of Medicine Taipei Taiwan; ^2^ Center of Genomic and Precision Medicine National Taiwan University College of Medicine Taipei Taiwan; ^3^ Department of Oncology National Taiwan University Hospital Taipei Taiwan; ^4^ Department of Medical Oncology National Taiwan University Cancer Center Taipei Taiwan

**Keywords:** bevacizumab, cetuximab, EGFR, metastatic colorectal cancer, panitumumab, secondary surgery

## Abstract

**Background:**

The first‐line systemic therapy for metastatic colorectal cancer (mCRC) is a combination of one targeted therapy agent and a chemotherapy doublet. Whether bevacizumab or anti‐epidermal growth factor receptor (anti‐EGFR) monoclonal antibody (mAb) is the more effective addition to a chemotherapy doublet as the first‐line treatment for inoperable *KRAS* wild‐type mCRC remains controversial in prior clinical trials. Moreover, the association between the sidedness of primary tumors and the efficacy of anti‐EGFR mAb needs to be addressed.

**Methods:**

We established a cohort of patients with *KRAS* wild‐type mCRC who were treated with first‐line targeted therapy plus doublet chemotherapy between 2013 and 2018 using Taiwan's National Health Insurance Research Database. Secondary surgery was defined as either resection of primary tumors, liver metastases, lung metastases, or radiofrequency ablation.

**Results:**

A total of 6482 patients were included; bevacizumab and anti‐EGFR mAb were the first‐line targeted therapies in 3334 (51.4%) and 3148 (48.6%) patients, respectively. Compared with those who received bevacizumab, patients who received anti‐EGFR mAb exhibited significantly longer overall survival (OS; median, 23.1 vs. 20.2 months, *p* = 0.012) and time to treatment failure (TTF; median, 11.3 vs. 10 months, *p* < 0.001). Among left‐sided primary tumors, the OS and TTF benefits of anti‐EGFR mAb remained. Among right‐sided primary tumors, the OS and TTF were similar regardless of the type of targeted therapy. In multivariate analyses, first‐line anti‐EGFR mAb therapy remained an independent predictor of longer OS and TTF for left‐sided primary tumors. Patients who received anti‐EGFR mAb were more likely to receive secondary surgery (29.6% vs. 22.6%, *p* < 0.0001) than patients who received bevacizumab.

**Conclusion:**

For patients who received first‐line doublet chemotherapy for *KRAS* wild‐type mCRC, adding anti‐EGFR mAb was associated with significantly longer OS and TTF, especially for left‐sided primary tumors.

## Graphical abstract

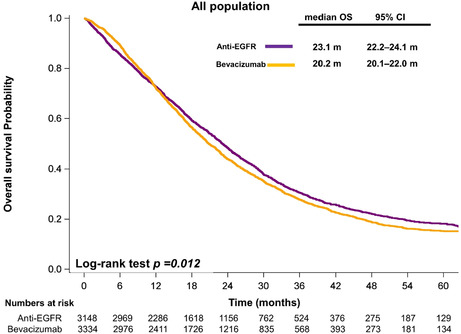

## 1 INTRODUCTION

Colorectal cancer (CRC) is one of most common cancers globally.^1^, ^2^ For inoperable metastatic colorectal cancer (mCRC), systemic therapy is the main treatment. The main frontline systemic chemotherapy agents include irinotecan, oxaliplatin, and 5‐fluorouracil. Doublet combination chemotherapy is usually recommended, such as irinotecan in combination with 5‐FU (FOLFIRI) or oxaliplatin in combination with 5‐FU (FOLFOX). The first‐line systemic therapy for mCRC is a combination of one targeted therapy agent and a chemotherapy doublet, such as FOLFIRI or FOLFOX, or a chemotherapy triplet in highly selected patients.^3^, ^4^ For *KRAS* wild‐type mCRC, if a chemotherapy doublet backbone is applied instead of a triplet, two types of targeted therapy agents can be considered in combination. One is antivascular endothelial growth factor (VEGF) monoclonal antibody (mAb) bevacizumab, and the other is anti‐epidermal growth factor receptor (anti‐EGFR) mAb such as cetuximab or panitumumab.

Regarding which targeted therapy is the more suitable companion of a chemotherapy doublet for *KRAS* wild‐type mCRC, randomized clinical trials have reported conflicting results. In the CALGB 80405 study, overall survival (OS) and progression‐free survival (PFS) were similar between patients who received bevacizumab or cetuximab in combination with a chemotherapy doublet.^5^, ^6^ In the FIRE‐3 study, patients who received cetuximab plus FOLFIRI had similar PFS but significantly longer OS compared with patients who received bevacizumab plus FOLFIRI.^7^, ^8^ By contrast, in the PEAK study, patients who received panitumumab plus FOLFOX had significantly longer PFS and OS compared with patients who received panitumumab plus FOLFOX.^9^, ^10^, ^11^ Furthermore, although the PFS was similar, patients who received panitumumab plus FOLFOX had significantly longer OS compared with patients who received panitumumab plus FOLFOX in the PARADIGM study.^12^ Therefore, whether anti‐VEGF mAb or anti‐EGFR mAb is suitable for incorporation in this first‐line systemic therapy remains unclear. Larger scale of clinical evidence is warranted to address this uncertainty on a realistic level.

Taiwan's National Health Insurance (NHI) program is a mandatory single‐payer system covering 97%–98% of the population.^13^ The associated database provided an opportunity to examine an unselected national population who had received fully reimbursed systemic anticancer therapies. Thus, we conducted this study to compare the treatment outcomes of first‐line targeted therapy agents added to a chemotherapy doublet as first‐line therapy for mCRC.

## 2 PATIENTS AND METHODS

### 2.1 Data source

In this retrospective population‐based cohort study, we linked data from three national databases, namely Taiwan's National Health Insurance Research Database for all inpatient and outpatient prescriptions, Taiwan Cancer Registry for patient demographics and disease status, and National Death Registry for death records. We retrieved data from Health and Welfare Data Science Center, Ministry of Health and Welfare, Taiwan. Detailed processes have been described in our previous studies.^14^, ^15^ In brief, all personal data extracted from these databases were encrypted and analyzed anonymously. Any results that applied to less than 3% of the targeted group were not processed to ensure privacy. The study was approved by the Institutional Review Board of National Taiwan University Hospital.

### 2.2 Study population and variables

All patients who fulfilled the following criteria were included in this study: (1) patients who received a diagnosis of histology‐proven primary CRC (ICD‐O‐3 C180 to C189, C19, and C20); (2) patients who were diagnosed as KRAS wild‐type mCRC; (3) patients who were aged 18 years or over; (4) patients who received first‐line targeted therapy of either bevacizumab, cetuximab, or panitumumab between January 1, 2013 and December 31, 2018, as the first‐line systemic therapy for *KRAS* wild‐type mCRC; and (5) received the targeted therapy in combination with either irinotecan or oxaliplatin. The exclusion criteria were as follows: (1) patients who had missing clinical data; (2) patients who had received a diagnosis of hematological malignancies or Kaposi's sarcoma (ICD‐O‐3 morphology code 9140, 9590–9989); (3) patients who received irinotecan and oxaliplatin at the same time; and (4) patients who had received another targeted agent before.

According to the targeted therapy agents used in the first‐line treatment regimen, patients were classified into the bevacizumab group and the anti‐EGFR mAb group. The index date was the date of the first dose of targeted therapy and chemotherapy. Secondary surgery referred to resection of primary tumors, liver metastases, or lung metastases or radiofrequency ablation to liver metastases after the index date. Left‐sided CRC was defined as the primary tumor origin in the rectum, sigmoid colon, descending colon, or splenic flexure. Right‐sided CRC was defined as the primary tumor origin in the cecum, ascending colon, hepatic flexure colon, or transverse colon. Elderly patients were defined as age ≧70 years old.^16^

### 2.3 Statistical analysis

OS was calculated from the index date to the date of death; the data were censored if patients survived beyond the last follow‐up date of December 31, 2019. The time to treatment failure (TTF) was calculated from the index date to the first dose of the next line of chemotherapy. Continuous variables expressed as mean were compared using the independent *t* test or Wilcoxon rank‐sum test, whereas categorical variables expressed as frequency was compared using the chi‐squared test or Fisher's exact test, as appropriate. OS and TTF were estimated using the Kaplan–Meier method, and the log‐rank test was employed to compare between‐group differences. The Cox proportional hazards model was applied to explore potential predictors of OS and TTF in multivariate analysis. Two‐sided *p* values <0.05 were considered statistically significant. All analyses were performed using SAS version 9.4 (SAS Institute, Cary, NC, USA).

## 3 RESULTS

### 3.1 Patient demographics

A total of 6482 patients were included in our study (Figure S1); 3334 (51.4%) of them received bevacizumab in combination with doublet chemotherapy, and 3148 (48.6%) received anti‐EGFR mAb (i.e., cetuximab or panitumumab) in combination with doublet chemotherapy. Over a median follow‐up of 18.6 months (95% confidence interval: 11.1–29.8 months), 4496 (69.4%) death events were observed.

Compared with those who received bevacizumab, the patients who received anti‐EGFR mAb as part of their first‐line systemic therapy were more likely to be men (*p* < 0.001), be aged 70 years or over (*p* < 0.001), undergo therapy at medical centers (*p* < 0.001), and have left‐sided primary tumors (*p* < 0.001); by contrast, they were less likely to have initial stage IV disease (*p* < 0.001), have adenocarcinoma histology (*p* < 0.001), and receive irinotecan as a part of the combination chemotherapy (*p* < 0.001; Table 1).

**TABLE 1:** Demographics of study patients, classified according to the first‐line targeted therapy agents received in combination with chemotherapy.

		Total (*N* = 6482)		Bevacizumab (*N* = 3334)		Anti‐EGFR mAb (*N* = 3148)		*p*
		*n*	(%)	*n*	(%)	*n*	(%)	
Gender	Male	3950	(60.9)	1958	(58.7)	1992	(63.3)	<0.001^b^
	Female	2532	(39.1)	1376	(41.3)	1156	(36.7)	
Treatment age	Median (min, max)	60	(18,92)	59	(19,92)	60	(18,92)	<0.001^b^
	<70	5071	(78.2)	2681	(80.4)	2390	(75.9)	<0.001^b^
	≥70	1411	(21.8)	653	(19.6)	758	(24.1)	
Hospital level	Medical center	3867	(59.7)	1910	(57.3)	1957	(62.2)	<0.001^b^
	Others	2615	(40.3)	1424	(42.7)	1191	(37.8)	
Primary site	Left	4931	(76.1)	2419	(72.6)	2512	(79.8)	<0.001^b^
	Right	1463	(22.6)	873	(26.2)	590	(18.7)	
	Others	88	(1.4)	42	(1.3)	46	(1.5)	
Initial stage	IV	4703	(72.6)	2658	(79.7)	2045	(65.0)	<0.001^b^
	I‐III	1779	(27.4)	676	(20.3)	1103	(35.0)	
Histology	Adenocarcinoma	6219	(95.9)	3227	(96.8)	2992	(95.0)	<0.001^b^
	Others	263	(4.1)	107	(3.2)	156	(5.0)	
Chemotherapy	Irinotecan	5990	(92.4)	3220	(96.6)	2770	(88.0)	<0.001^b^
	Oxaliplatin	492	(7.6)	114	(3.4)	378	(12.0)	
	5‐FU combination^a^	5932	(91.5)	3037	(91.1)	2895	(92.0)	0.208
	Tegafur combination	92	(1.4)	40	(1.2)	52	(1.7)	0.124
	Capecitabine	241	(3.7)	137	(4.1)	104	(3.3)	0.087

Abbreviations: EGFR, epidermal growth factor receptor; mAb, monoclonal antibody; 5‐FU, 5‐fluorourocil.

*Note*: All data are presented as *N* (%) unless indicated otherwise.

^a^ Patients may shift between 5‐FU and its analogs.

^b^ *p* < 0.05.

### 3.2 Treatment outcomes

**FIGURE 1:**
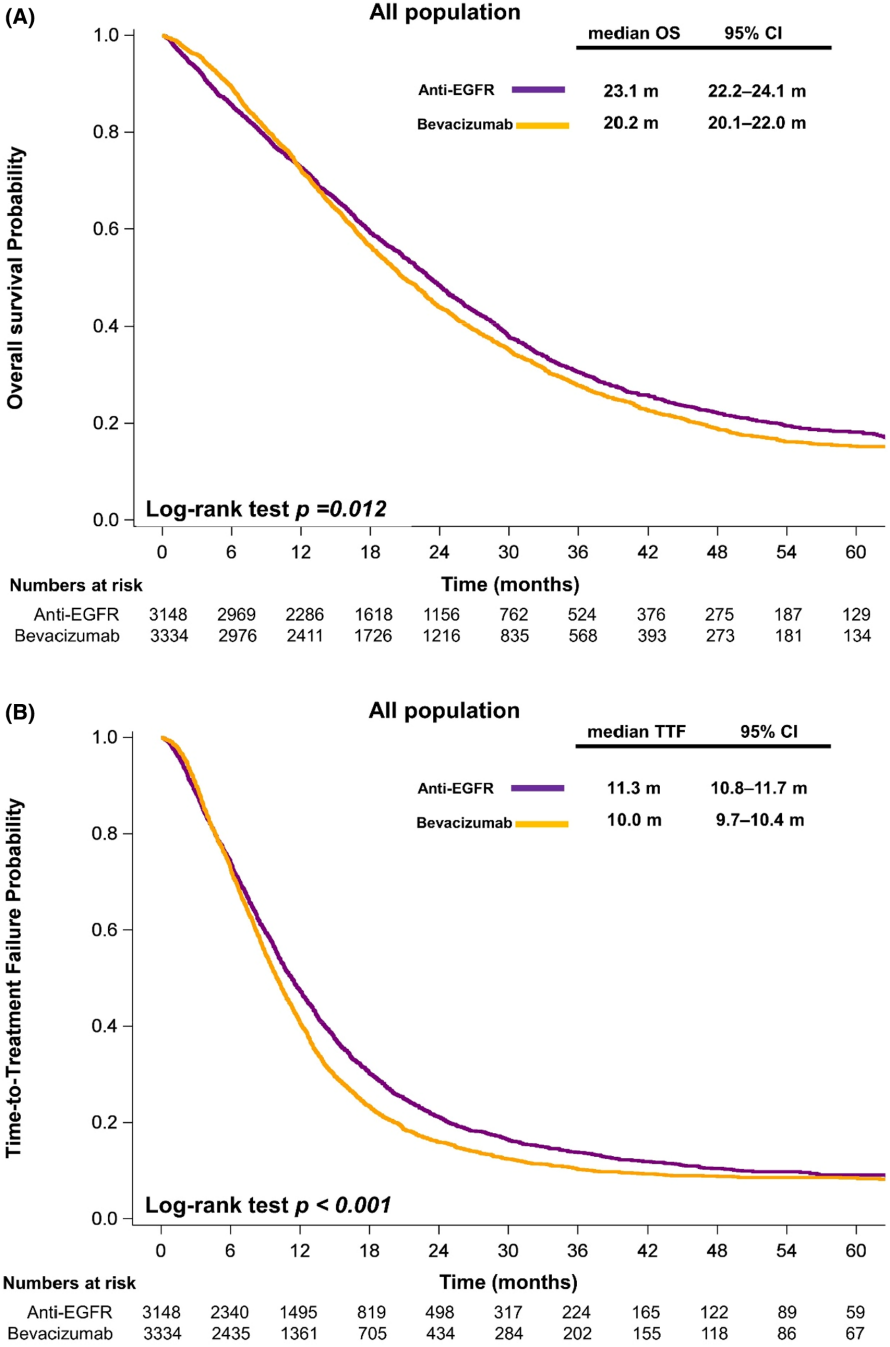
Kaplan–Meier plots illustrating (A) overall survival and (B) time to treatment failure according to the treatment regimen. *p* values were determined using the log‐rank test. OS, overall survival; TTF, time to treatment failure; anti‐EGFR, anti‐epidermal growth factor receptor.

Compared with the patients who received bevacizumab, the patients who received anti‐EGFR mAb in their first‐line systemic therapy exhibited significantly longer OS (median, 23.1 vs. 20.2 months, *p* = 0.012; Figure 1A) and TTF (median, 11.3 vs. 10 months, *p* < 0.001; Figure 1B).

**FIGURE 2:**
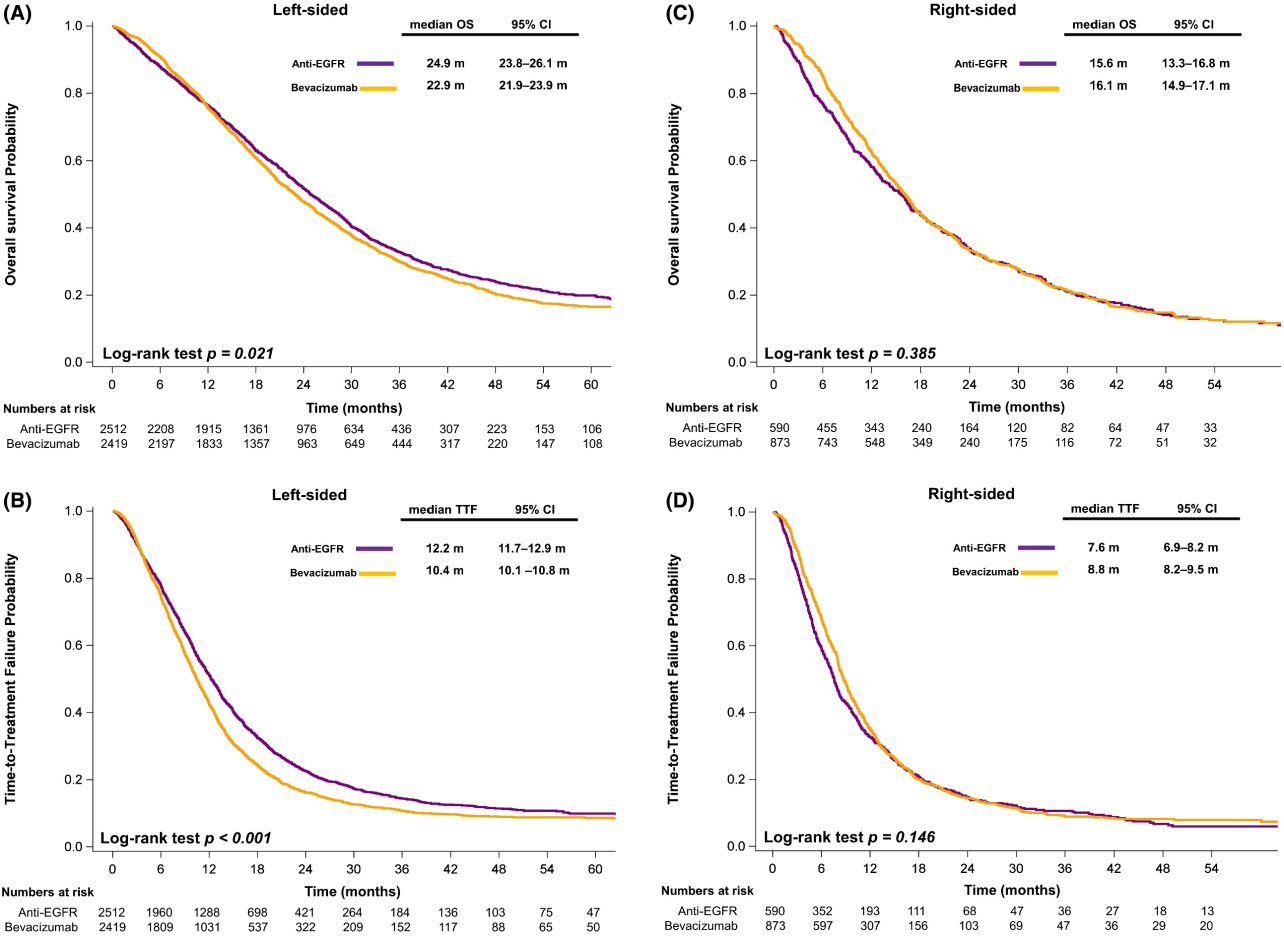
(A–B) Kaplan–Meier plots illustrating (A) overall survival and (B) time to treatment failure according to the treatment regimens of patients with left‐sided primary tumors; (C–D) Kaplan–Meier plots illustrating (C) overall survival and (D) time to treatment failure according to the treatment regimens of patients with right‐sided primary tumors. *p* values were determined using the log‐rank test. OS, overall survival; TTF, time to treatment failure; anti‐EGFR, anti‐epidermal growth factor receptor.

We further analyzed the treatment outcomes according to the sidedness of the primary tumor. Among the patients with left‐sided mCRC, those who received anti‐EGFR mAb in first‐line systemic therapy exhibited significantly longer OS (median, 24.9 vs. 22.9 months, *p* = 0.021; Figure 2A) and TTF (median, 12.2 vs. 10.4 months, *p* < 0.001; Figure 2B) compared with those who received bevacizumab. Among the patients with right‐sided mCRC, those who received either anti‐EGFR mAb or bevacizumab in their first‐line systemic therapy exhibited similar OS (median, 15.6 vs. 16.1 months, *p* = 0.385; Figure 2C) and TTF (median, 7.6 vs. 8.8 months, *p* = 0.146; Figure 2D).

### 3.3 Multivariate analysis

In the multivariate analyses of the entire cohort data with adjustment for age, gender, sidedness of the primary tumor, initial CRC stage, histology, combination chemotherapy, and hospital level, receiving anti‐EGFR mAb or bevacizumab in combination with first‐line chemotherapy was not associated with OS (hazard ratio [HR] = 0.96, *p* = 0.195). By contrast, the addition of anti‐EGFR mAb as the first‐line targeted therapy remained an independent predictor of longer TTF (HR = 0.91, *p* < 0.001; Table 2, Table S1). In addition, right‐sided primary tumor and initial stage IV disease were independent predictors of poor OS and TTF (Table S1).

**TABLE 2:** Cox proportional hazards models for potential predictors of overall survival (OS) and time to treatment failure (TTF).

Variable	OS			TTF		
	HR	(95% CI)	p	HR	(95% CI)	p
Anti‐EGFR mAb (vs. bevacizumab)	0.96	(0.90–1.02)	0.195	0.91	(0.86–0.96)	<0.001^a^
Age ≥ 70 years	1.35	(1.27–1.45)	<0.001^a^	1.05	(0.99‐1.12)	0.121
Female	0.93	(0.88–0.99)	0.023^a^	0.98	(0.93‐1.03)	0.413
Left‐sided (vs. right‐sided)	0.70	(0.65–0.75)	<0.001^a^	0.79	(0.74‐0.84)	<0.001^a^
Initial stage IV	1.17	(1.09–1.25)	<0.001^a^	1.26	(1.19‐1.34)	<0.001^a^
Adenocarcinoma histology (vs. others)	0.94	(0.81–1.09)	0.425	0.95	(0.83–1.08)	0.432
Combination with oxaliplatin (vs. irinotecan)	1.00	(0.89–1.13)	0.994	1.14	(1.03–1.26)	0.014^a^
Medical center (vs. others)	0.97	(0.91–1.03)	0.271	1.01	(0.96–1.07)	0.675

Abbreviations: CI, confidence interval; EGFR, epidermal growth factor receptor; HR, Hazard ratio; mAb, monoclonal antibody; OS, overall survival; TTF, time to treatment failure.

^a^ *p* < 0.05.

Because the sidedness of the primary tumor was associated with the choice of first‐line targeted therapy, OS, and TTF (Tables 1 and 2), we performed separate multivariate analyses for the patients with left‐sided and right‐sided primary tumors. For the patients with left‐sided primary tumors, first‐line anti‐EGFR mAb was an independent predictor of OS (HR = 0.93, *p* = 0.030) and TTF (HR = 0.84, *p* < 0.001; Table 3). For the patients with right‐sided primary tumors, first‐line anti‐EGFR mAb was not associated with OS (*p* = 0.270) but was an independent predictor of shorter TTF (HR = 1.12, *p* = 0.047; Table 3). Initial stage IV disease was an independent predictor of poor OS and TTF, regardless of the sidedness of primary tumors (Table S1).

**TABLE 3:** Cox proportional hazards models for potential predictors of overall survival and time to treatment failure, in left‐sided and right‐sided primary tumors, respectively.

Variable	Overall Survival						Time to treatment failure					
	Left‐sided			Right‐sided			Left‐sided			Right‐sided		
	HR	(95% CI)	*p*	HR	(95% CI)	*p*	HR	(95% CI)	*p*	HR	(95% CI)	*p*
Anti‐EGFR mAb (vs. bevacizumab)	0.93	(0.86–0.99)	0.030^a^	1.07	(0.95–1.21)	0.270	0.84	(0.79–0.90)	<0.001^a^	1.12	(1.00–1.26)	0.047^a^
Age ≥ 70 years	1.43	(1.32–1.56)	<0.001^a^	1.18	(1.04–1.35)	0.011^a^	1.10	(1.02‐1.18)	0.017^a^	0.95	(0.84–1.08)	0.419
Female	0.89	(0.83–0.96)	0.002^a^	1.05	(0.94–1.18)	0.388	0.96	(0.90–1.02)	0.203	1.05	(0.94–1.17)	0.366
Initial stage IV	1.13	(1.04–1.22)	0.003^a^	1.26	(1.10–1.45)	0.001^a^	1.25	(1.16–1.34)	<0.001^a^	1.27	(1.12–1.46)	<0.001^a^
Adenocarcinoma histology (vs. others)	0.82	(0.69–0.97)	0.022^a^	1.27	(0.93–1.73)	0.132	0.87	(0.75–1.02)	0.082	1.16	(0.87–1.53)	0.307
Combination with oxaliplatin (vs. irinotecan)	0.98	(0.85–1.14)	0.821	1.04	(0.82–1.32)	0.737	1.14	(1.01–1.28)	0.037^a^	1.11	(0.90–1.38)	0.331
Medical center (vs. others)	0.97	(0.90–1.04)	0.368	0.97	(0.86–1.09)	0.605	1.02	(0.96–1.08)	0.550	0.99	(0.89–1.11)	0.920

Abbreviations: CI, confidence interval; EGFR, epidermal growth factor receptor; HR, Hazard ratio; mAb, monoclonal antibody.

^a^ *p* < 0.05.

### 3.4 Secondary surgery

**FIGURE 3:**
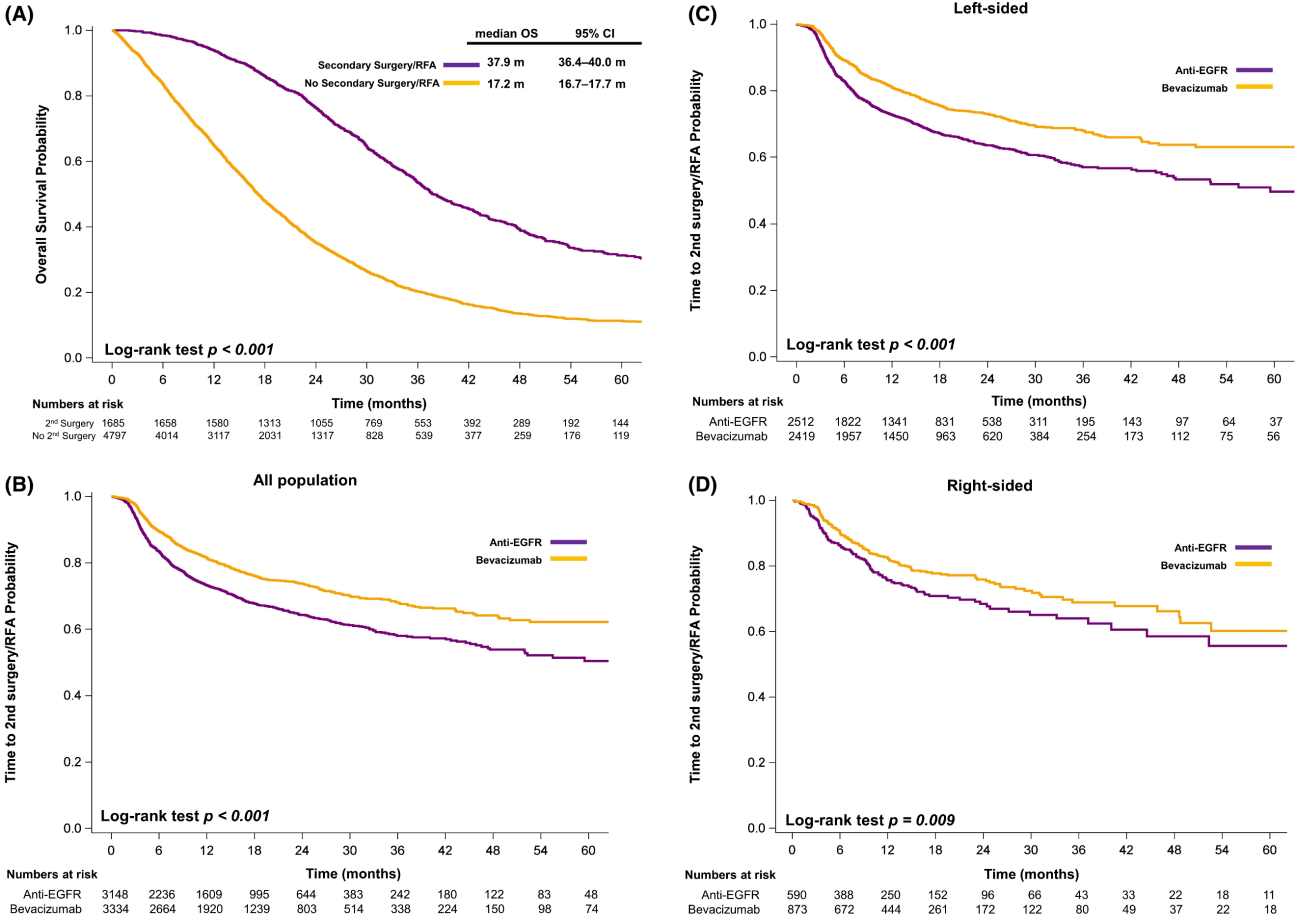
(A) Kaplan–Meier plots illustrating the overall survival of patients according to whether they received secondary surgery or radiofrequency ablation (RFA); (B–D) Kaplan–Meier plots illustrating time to secondary surgery or RFA according to the treatment regimens of the (B) whole population, (C) patients with left‐sided primary tumors, and (D) patients with right‐sided primary tumors. *p* values were determined using the log‐rank test. OS, overall survival; anti‐EGFR, anti‐epidermal growth factor; 2nd, secondary.

Among the 6482 patients in this study, 1685 (26.0%) received secondary surgery after the initiation of first‐line systemic therapy. The patients who received secondary surgery exhibited significantly longer OS than the patients who did not (median, 37.9 vs. 17.2 months, *p* < 0.001; Figure 3A). The patients who received anti‐EGFR had a significantly higher chance of undergoing secondary surgery (29.6% vs. 22.6%, *p* < 0.001) and a significantly shorter time to secondary surgery compared with the patients who received bevacizumab (*p* < 0.001, Figure 3B). Among the patients who received secondary surgery, the median time to secondary surgery was 5.7 months in the anti‐EGFR mAb group and 7.1 months in the bevacizumab group.

For the patients with left‐sided primary tumors, those who received anti‐EGFR had a significantly higher chance of undergoing secondary surgery (31.1% vs. 23.6%, *p* < 0.001) and a significantly shorter time to secondary surgery compared with those who received bevacizumab (Figure 3C). Among the patients with left‐sided primary tumors who received secondary surgery, the median time to secondary surgery was 5.7 months in the anti‐EGFR mAb group and 7.2 months in the bevacizumab group.

For the patients with right‐sided primary tumors, those who received anti‐EGFR had a higher chance of undergoing secondary surgery (23.4% vs. 19.9%, *p* = 0.113) and a significantly shorter time to secondary surgery compared with those who received bevacizumab (Figure 3D). Among the patients with right‐sided primary tumors who underwent secondary surgery, the median time to secondary surgery was 5.8 months in the anti‐EGFR mAb group and 6.7 months in the bevacizumab group.

## 4 DISCUSSION

In this nationwide cohort study, we demonstrated that among patients who received first‐line chemotherapy doublets for inoperable *KRAS* wild‐type mCRC, the addition of anti‐EGFR mAb led to significantly longer OS and TTF compared with the addition of bevacizumab. This benefit was mainly observed in the patients with left‐sided primary tumors. In the multivariate analysis, anti‐EGFR mAb treatment remained an independent predictor of longer OS and TTF among those with left‐sided primary tumors. To the best of our knowledge, this is the largest (*n* = 6482) cohort study focusing on this topic.

Our study results are compatible with those of the FIRE‐3 and PARADIGM randomized clinical trial studies, which have demonstrated that patients who receive anti‐EGFR mAb exhibit significantly longer OS than patients who receive bevacizumab in combination with a chemotherapy doublet.^7^, ^8^, ^9^, ^10^, ^11^, ^17^ However, the phase III CALGB 80405 study reported similar OS between patients who received either bevacizumab or cetuximab as the combination targeted therapy agents.^5^, ^6^, ^9^ A pooled analysis of these three studies indicated that anti‐EGFR mAb as the first‐line targeted agent for *RAS* wild‐type mCRC significantly benefitted OS, especially in patients with left‐sided mCRC.^18^ In Taiwan, our recent treatment consensus also addressed the importance of first‐line anti‐EGFR mAb in combination with the chemotherapy doublet, especially for left‐sided tumors and for therapy with curative intent.^19^

The association between the sidedness of primary tumors and the efficacy of anti‐EGFR mAb has been reported in many unplanned subgroup analyses of randomized studies.^9^, ^20^, ^21^, ^22^ A meta‐analysis of 12 randomized studies with propensity score matching verified this association^23^; however, this study did not include the aforementioned FIRE‐3 and PEAK studies. Thus, it was not surprising that the study did not reveal anti‐EGFR mAb to be more effective than bevacizumab as the companion targeted therapy in first‐line systemic treatment for left‐sided *KRAS* wild‐type mCRC. In another meta‐analysis of six main clinical studies including FIRE‐3 and PEAK studies, the benefit of anti‐EGFR mAb for left‐sided *KRAS* wild‐type mCRC was disclosed.^24^ Currently, anti‐EGFR mAb is suggested with the preferred regimen for left‐sided *KRAS* wild‐type mCRC in most updated guidelines.^25^, ^26^

We determined several possible reasons for the survival benefit of first‐line anti‐EGFR mAb, especially in patients with left‐sided primary tumors, in a single population without the need for combining multiple cohorts. We studied an unselected nationwide cohort with a large sample size through multivariate analysis. In addition, over 90% of the patients received irinotecan as the companion chemotherapy instead of oxaliplatin. The results of several clinical trials revealed that irinotecan‐based doublet chemotherapy in combination with targeted therapy led to longer PFS and OS, although these were nonsignificant, than oxaliplatin‐based doublet chemotherapy.^27^, ^28^, ^29^ These results imply that irinotecan might exert more synergistic effects in combination with targeted therapy than oxaliplatin.^30^

Whether anti‐EGFR mAb can be considered a first‐line targeted therapy option for patients with right‐sided primary tumors remains controversial. In the National Comprehensive Cancer Network guideline, its use is not favored in patients with expanded *RAS* wild‐type right‐sided mCRC.^31^ Contrarily, in the pan‐Asian adapted European Society for Medical Oncology guideline, anti‐EGFR mAb plus a chemotherapy doublet is as an option for patients who were unsuitable for bevacizumab plus a chemotherapy triplet.^4^ In our data, among the patients with right‐sided primary tumors, those who received bevacizumab exhibited significantly longer TTF than those who received anti‐EGFR mAb, but this difference in TTF did not translate to longer OS. Our data support the use of anti‐EGFR mAb as one of the first‐line options for targeted therapy for right‐sided mCRC, which is in line with a consensus guideline in Taiwan.^19^

Secondary surgery after first‐line treatment for mCRC provides a crucial chance of cure.^8^, ^32^ When combined with doublet chemotherapy, anti‐EGFR mAb resulted in more frequent early tumor shrinkage and a deeper objective tumor response than bevacizumab,^33^ which could lead to a higher secondary resection rate. However, in the PEAK study, the secondary resection rate was 14.7% and 16.2% in the panitumumab and bevacizumab arms, respectively.^34^ In the DEEPER trial, cetuximab in combination with triplet chemotherapy induced significantly deeper tumor responses compared with bevacizumab plus a chemotherapy triplet^35^; however, the secondary resection rate was similar between the two arms. Furthermore, in the PARADIGM study, the secondary resection rate was 16.5% and 10.9% in the panitumumab and bevacizumab arms, respectively.^17^ Our study indicated that anti‐EGFR mAb plus chemotherapy led to significantly higher secondary resection rates, regardless of the primary tumor location, which was compatible with the results in the PARADIGM study. Because of the retrospective nature of our study, we could not adjust for other critical confounding factors, such as tumor size and metastatic site. Whether anti‐EGFR mAb results in a higher secondary surgery rate must be further explored.

Our current study had some limitations. First, the data of prescriptions not reimbursed through NHI were unavailable, including those of second‐line bevacizumab treatment or immunotherapy paid for by patients themselves. However, our data reflected the actual scenario when second‐line targeted therapy was not reimbursed. Second, information on genetic aberrations other than *KRAS* mutations, such as *BRAF* and DNA mismatch repair status, was unavailable. Instead, our data implied that even in the population that exhibited the *NRAS* or *BRAF* mutation, bevacizumab as a first‐line targeted therapy did not offer greater survival benefits over anti‐EGFR mAb. Immunotherapy was proven to be beneficial for microsatellite instability‐high colorectal cancer in multiple clinical trials,^36^, ^37^, ^38^ but the enrollment period of our study was before the publication of these results. Many genetic alterations were found to be predictive of the efficacy of specific therapies, such as *HER2* amplification and tumor mutation burden.^39^ However, the evidence of these genetic alterations were for salvage therapy rather than first‐line therapy. Finally, as mentioned, we could not verify whether the disease extent before systemic therapy differed between the anti‐EGFR mAb group and bevacizumab group.

In conclusion, for the patients who received first‐line doublet chemotherapy for *KRAS* wild‐type mCRC, the addition of anti‐EGFR mAb was associated with significantly longer OS and TTF than the addition of bevacizumab, especially for those with left‐sided primary tumors.

## AUTHOR CONTRIBUTIONS

**Yi‐Hsin Liang:** Conceptualization (equal); funding acquisition (equal); investigation (equal); methodology (equal); project administration (equal); resources (equal); writing – original draft (equal). **Kuo‐Hsing Chen:** Conceptualization (equal); validation (equal); writing – review and editing (equal). **Yu‐Yun Shao:** Resources (equal); software (equal); supervision (equal); validation (equal); visualization (equal); writing – original draft (equal); writing – review and editing (equal).

## FUNDING INFORMATION

This study was supported by Ministry of Science and Technology, Taiwan (MOST 105‐2314‐B‐002‐194, MOST 106‐2314‐B‐002‐213, MOST 108‐2314‐B‐002‐072‐MY3, and MOST 111‐2314‐B‐002‐120); National Taiwan University Hospital, Taipei, Taiwan(NTUH 105‐S2954, NTUH 108‐S4150); and the Science and Technology Unit, Ministry of Health and Welfare, Taiwan (DOH102‐NH‐9002 and MOHW112‐TDU‐B‐211‐144‐002).

## CONFLICT OF INTEREST STATEMENT

All authors claim that there is no any conflict of interest.

## ETHICS STATEMENT

All results in our study were approved by the Institutional Review Board of National Taiwan University Hospital (Certification number: NTUH#202206062 W).

All personal data were encrypted and analyzed anonymously.

## Supporting information


Appendix S1

## Data Availability

These three national databases were stored separately and are available only to researchers who officially apply for access. Requests for copies of the data set can be made directly to the authoritative body.^40^, ^41^
