# Supplementary material for: Treatment outcome comparisons of first‐line targeted therapy in patients with KRAS wild‐type metastatic colorectal cancer: A nationwide database study
Source: Cancer Med. 2023 Jun 16;12(14):15176–86. doi: 10.1002/cam4.6196 (PMC10417087; doi:10.1002/cam4.6196)
Supplement: Supplementary file 1 — Appendix S1 [file CAM4-12-15176-s001.docx]

**Figure S1.** Patient flow diagram. EGFR: epidermal growth factor receptor; mAb: monoclonal antibody

Exclude patients:

- without pathology confirmation (n=1,976)
- age less than 18 years old (n=30)
- no prescription of bevacizumab, cetuximab or panitumumab in 2013-2018 (n=86,803)
- prescription of bevacizumab or cetuximab before 2013 (n=4,991)

Patients with newly diagnosed colon cancer between 2011-2018,

N=111,854

Patients who received standard 1^st^ line regimen for mCRC, N=15,769

Bevacizumab (n=12,621)

Cetuximab (n=2,868)

Panitumumab (n=280)

Exclude patients:

- prescription of irinotecan before (index date-3m) (n=739)
- Without concurrently use of irinotecan or oxaliplatin (n=1,063)
- Both of Irinotecan and oxaliplatin were prescribed concurrently (n=483)

Study patients, known *KRAS* wild type, N=6,482

Bevacizumab (n=3,334)

Anti-EGFR mAb (n=3,148)

Patients who had first prescription of bevacizumab, cetuximab or panitumumab during 2013 and 2018,

N=18,054

**Table S1**. Cox proportional hazards models for potential predictors of overall survival (OS) and time to treatment failure (TTF)

| **Variable** | **OS** | | |  | **TTF** | | |
| --- | --- | --- | --- | --- | --- | --- | --- |
|  | HR | (95% CI) | *p* |  | HR | (95% CI) | *p* |
| **Anti-EGFR mAb (vs. bevacizumab)** | 0.96 | (0.91-1.02) | 0.208 |  | 0.91 | (0.86-0.96) | <0.001^*^ |
| **Age** |  |  |  |  |  |  |  |
| <40 | ref |  | <0.001* |  | ref |  | <0.001* |
| 40-50 | 0.81 | (0.70-0.92) |  |  | 0.83 | (0.74-0.94) |  |
| 50-60 | 0.78 | (0.69-0.88) |  |  | 0.83 | (0.74-0.93) |  |
| 60-70 | 0.80 | (0.70-0.90) |  |  | 0.79 | (0.70-0.88) |  |
| 70-80 | 1.02 | (0.90-1.16) |  |  | 0.84 | (0.75-0.95) |  |
| > 80 | 1.46 | (1.24-1.72) |  |  | 0.98 | (0.84-1.14) |  |
| **Female** | 0.93 | (0.87-0.98) | 0.0134^*^ |  | 0.97 | (0.92-1.02) | 0.223 |
| **Left-sided (vs. right-sided)** | 0.70 | (0.66-0.75) | <0.001^*^ |  | 0.79 | (0.74-0.84) | <0.001^*^ |
| **Initial stage IV** |  |  |  |  |  |  |  |
| I-II | ref |  | <0.001^*^ |  | ref |  | <0.001^*^ |
| III | 1.17 | (1.02-1.34) |  |  | 1.10 | (0.98-1.25) |  |
| IV | 1.31 | (1.16-1.49) |  |  | 1.35 | (1.21-1.51) |  |
| **Adenocarcinoma histology (vs. others)** | 0.93 | (0.80-1.08) | 0.335 |  | 0.95 | (0.83-1.08) | 0.421 |
| **Combination with oxaliplatin (vs. irinotecan)** | 0.99 | (0.87-1.12) | 0.824 |  | 1.14 | (1.03-1.26) | 0.015^*^ |
| **Medical center (vs. others)** | 0.97 | (0.91-1.03) | 0.247 |  | 1.01 | (0.96-1.07) | 0.673 |
| Abbreviations: OS: overall survival; TTF: time to treatment failure; HR: Hazard ratio; CI: confidence interval; EGFR: epidermal growth factor receptor; mAb: monoclonal antibody  ^*^*p* <0.05 | | | | | | | |
